# Comparative Study on the Biochemical Profile and Antioxidant Activity of *Picrorhiza kurrooa* Rolye ex Benth. Obtained from Uttarakhand

**DOI:** 10.1155/2023/8792414

**Published:** 2023-12-07

**Authors:** Anshika Pokhriyal, Soban Prakash, Babita Patni

**Affiliations:** High Altitude Plant Physiology Research Centre, Hemvati Nandan Bahuguna Garhwal University (A University), Srinagar Garhwal, Uttarakhand, India

## Abstract

*Picrorhiza kurrooa* Royle ex Benth. is one of the well-established herbal plants with an exceptional therapeutic potential. It belongs to the Scrophulariaceae family and is commonly called as kutki. The drug obtained from the plant is a bitter tonic due to the presence of kutkin in it. Over 61 secondary metabolites from the plants have been identified, including iridoid glycosides, flavonoids, cucurbitacins, and phenolic chemicals. However, picrosides are the major phytochemicals in this species that are responsible for its well-known hepatoprotective properties. The present study was conducted to compare *Picrorhiza kurrooa* (dried rhizomes) obtained from local traders from the markets of three different districts of Uttarakhand, i.e., the Dewal block of Chamoli, Ukhimath block of Rudraprayag, and Dharchula block of Pithoragarh. Biochemical analysis was conducted on the powder of dried rhizomes for alkaloids, phenolics, tannins, flavonoids, and antioxidant activity. Based on analysis, it was found that the total phenolic content, total flavonoid content, total alkaloid content, and radical scavenging activity of *P*. *kurrooa* rhizomes purchased from Darma valley, Pithoragarh district of Uttarakhand, were the highest, followed by rhizomes collected from the Dewal block of Chamoli district and the least were found in rhizomes obtained from the Ukhimath block of Rudraprayag district of Uttarakhand. The maximum tannin content was found in *Picrorhiza kurrooa* rhizomes obtained from the Dewal block of Chamoli, while total reducing power was observed the highest in rhizomes from the Ukhimath block of Rudraprayag. The results provided evidence that *P. kurrooa* obtained from Darma valley, Pithoragarh, are the potential source of phenolics, flavonoids, and tannins and have the highest DPPH-scavenging activity and therefore could be served as the basis for future drugs and food materials.

## 1. Introduction

Due to its special location inside the Himalayan region, Uttarakhand boasts luxuriant and diverse vegetation. Almost every plant has economic worth from a nutritional, aesthetic, or medical standpoint. This Himalayan region supplies a sizable portion of the crude pharmaceuticals sold in India [1].

There are a huge number of noteworthy medicinal plants around the world that are of tremendous value in traditional medical systems. One such major high-altitude medicinal plant is *Picrorhiza kurrooa* Royle ex Benth. The family Scrophulariaceae includes the genus Picrorhiza, which is well known for its therapeutic properties. This family includes two significant, endangered medicinal plant species, *P. kurrooa* Royle ex Benth. and *P. scrophulariiflora* Pennel, which are native to India, Nepal, Tibet, Pakistan, and China [2]. *P. kurrooa* (kutki, the local name) predominates in the western Himalayas of northern Indian, whereas *P. scrophulariiflora* is mostly found in the Himalayan regions of Sikkim, Tibet, and Nepal.

The widespread use of medicinal plants from their natural habitats has resulted in a significant depletion in their numbers. This, coupled with the absence of structured cultivation methods, has resulted in these species being classified as endangered by the International Union for Conservation of Nature and Natural Resources (IUCN) [3, 4].

The bitter root of *Picrorhiza*, which is employed as a traditional medicinal, gave rise to the generic name of the plant [4]. The word *Picrorhiza* is made up of two words, the Greek word “picross” which denotes a meaning bitter, while the word “rhiza” denotes roots [5]. The common name “kutki” comes from the Punjabi name of the plant, “karu,” which means bitter [6].

**Figure 1:**
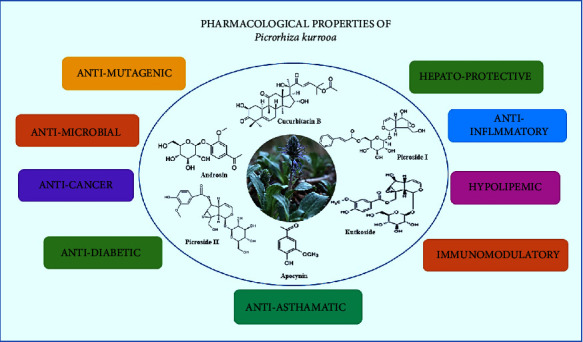
Major phytochemicals and pharmacological properties of *P. kurrooa*.

It is a plant with significant medicinal and pharmacological use. It possesses various pharmacological properties, including hepatoprotective [7, 8], antiasthma, anti-inflammatory, antidiabetic [9, 10], antimicrobial, antimutagenic, antimalarial, cardioprotective, anticancer [11], antiulcer, and neuroprotective and antioxidant activities [12]. The antioxidant properties have been reported in the *P. kurrooa* rhizome extract. The extract's ability to scavenge oxygen-free radicals and prevent lipid peroxidation [13] is mostly attributable to the presence of flavonoid and phenolic components in it [14, 15]. These medicinal properties are attributed to the phytochemical constituents present in *P. kurrooa* (depicted in Figure 1).

### 1.1. Biochemical Constituents in *P. kurrooa*

According to the study's data on its phytochemistry, it has a complex variety of phytochemicals. It primarily contains cucurbitacins, acetophenones, and iridoids [16]. Sah and Varshney have reported 61 biochemical compounds from different parts of *P. kurrooa* [17]. The active biochemical present in *P. kurrooa* is kutkin (a bitter constituent), which is made up of kutkosides and picrosides. Picroside I and picroside II, that are ideally iridoid glycosides, are the two main picrosides found in kutkin [18]. Other iridoid glycosides include picroside III, picroside IV, picroside V, veminoside, catalpol, veronicoside, specioside, minecoside, picrorhizaoside, 6-feruloylcatalpol, pikuroside, and aucubin [19]. Iridoid gylcosides are considered responsible for hepatoprotective properties of the plant. Other constituents present in this plant are cucurbitacins (B, D, and R) [20], minecoside, picein, pikuroside, flavonoid apocyanin, vanillic acid, ferullic acid, D-mannitol [19, 21], a ketone kutkiol, kutkisterol, and phenolic glycosides such as apocyanin (4′-hydroxy-3′-methoxy acetophenone), androsin, picein [22, 23], triterpenoids (in seeds) [17], lignan, coumarin, isoflavans, porphyrins, terpene lactone [24], and phenylethanoid glycosides (kurroaoside) [25]. Cucurbitacins are renowned for their antitumor and cytotoxic attributes. Apocynin serves as a potent inhibitor of NADPH (nicotinamide adenine dinucleotide phosphate) oxidase, displaying anti-inflammatory and antioxidant characteristics. In addition, androsin is known for its antiasthmatic properties [26].

### 1.2. Morphology

It is a perennial herb with stolons that are elongated, sturdy, and creeping type and are covered with withered leaf bases. The stem is small, weak, creeping, slightly hairy, leafy, and erect at the flowering end [27]. Leaves are 5–10 cm long, sharply serrate, almost radical, spathulate, and base narrowed with winged sheathing petiole [28]. Inflorescence is racemose. Flowers are very small and white or pale blue-purple coloured [27–29]. Fruits are an ovoid capsule; each plant contains 5–14 capsules, with each having 32–65 seeds. Seeds are small without seed coat. The roots of this plant always feature transverse cracks and longitudinal wrinkles [30]. The rhizome is cylindrical, irregularly curved, 2.5–8 cm long, 4–8 mm thick, externally grayish brown in colour, jointed, and zigzag with jointed nodes having branching and rooting at them. The rhizomes and roots have a bitter taste [16].

### 1.3. Distribution

It is a significant alpine herb that grows between 3000 m and 5000 m above the mean sea level in the Himalayan area. Although this plant normally grows at elevations greater than 3000 masl, it may also be grown at lower elevations [27, 29, 31]. It is native to western Himalayas and extends up to the mountains of Yunnan in China [32]. In Uttarakhand, it is naturally found in areas of Uttarkashi (Bhatwari, Har-ki-Dun, and Raiwan valley) [33, 34], Chamoli (Ghes and Badrinath) [35], Rudraprayag (Kedarnath, Tungnath, and Madmaheswar) [36], Pithoragarh (Kutti, Gunji, Ralam, Ganghar, LaspaNapalchunala, and Panchachuli), and Bageshwar (Devikund, Sunderdunga, Dwali, Phurkia, and Pindari) districts [37]. In Himachal Pradesh, it is found in the higher reaches of Shimla, Kullu, Kinnaur, Kangra, Chamba, Mandi, Lahaul, and Spitivalley [38]. In Kashmir Himalayas, it grows in high reaches of Gurez valley, Lolab, Keran, Sindh, and Liidder valleys [16]. It is commonly seen associated with the herbs such as *Potentilla kashmirica*, *Latotiscashmiriana*, *Aconitum violaceum*, *Senecio jacquemontianus*, and *Sedum ewersii* [39].

*P. kurrooa* is a long-creeping plant that thrives in soils with high levels of organic matter and with sandy, clay loamy in texture [35]. It prefers slopes with a north-west orientation that are moist and generally less exposed [16].

The International Union for Conservation of Nature and Natural Resources (IUCN) has designated the species as endangered due to its overexploitation and a lack of systematic cultivation [2, 3].

This study was conducted keeping in view the following two objectives:

1. To compare and validate the medicinal and nutritional potential of *Picrorhiza kurrooa* rhizomes obtained from different districts of Uttarakhand.
2. To assess the variations in the chemical composition and antioxidant activity of *Picrorhiza kurrooa* rhizome collected from three local traders of different high-altitude habitats of Uttarakhand, India, to get knowledge about the level of authenticity of the plant material.

## 2. Materials and Methodology

### 2.1. Collection of Plant Material

Plant materials (dried roots and rhizome) were purchased from local traders only after confirming the source of purchase. The samples acquired from the Dewal block's market in Chamoli were obtained from Ghes village. Similarly, the sample obtained from the market of Darma valley in Pithoragarh was collected from Gunji village, whereas the sample from the Ukhimath block in Rudraprayag district was sourced from cultivable fields located in Pothiwasa. A voucher specimen has been deposited in HAPPRC, Srinagar Garhwal, Uttarakhand, against voucher no. HAPPRC2309.

**Figure 2:**
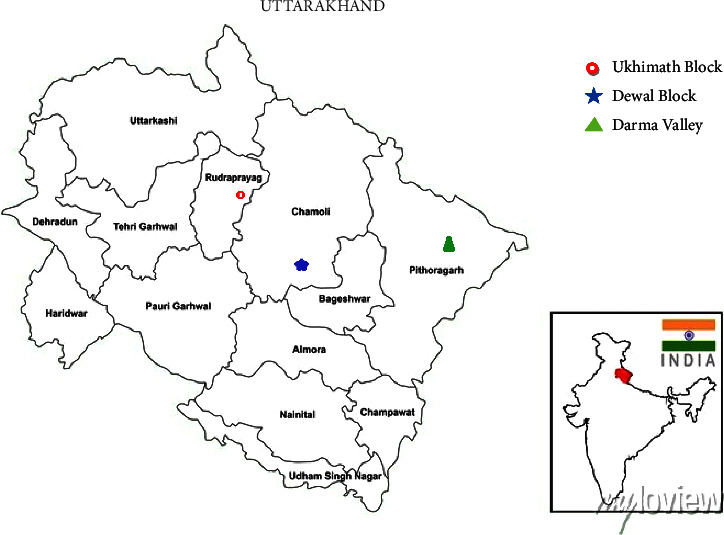
Study area; sites from where *Picrorhiza kurrooa* rhizomes were obtained.

**Figure 3:**
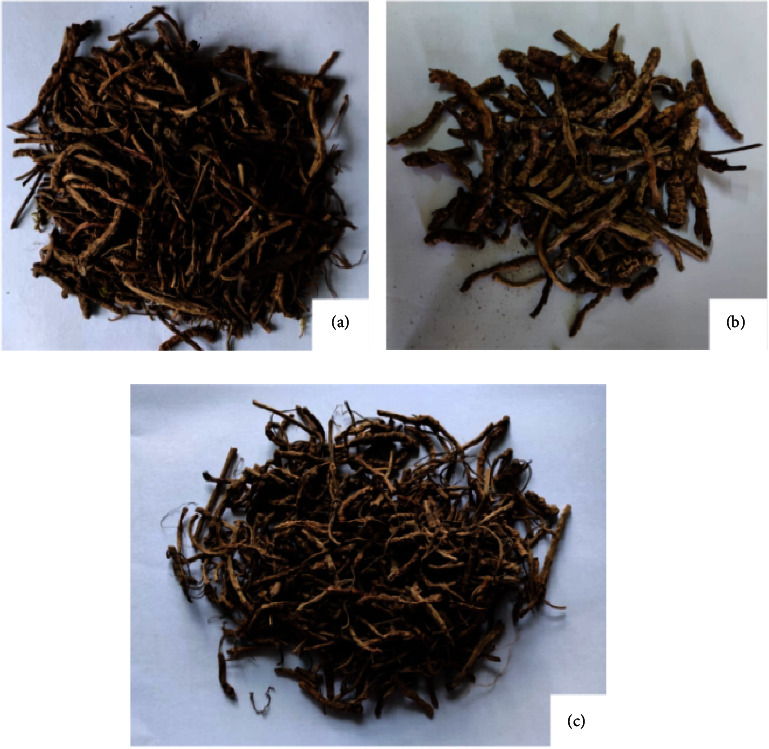
Dried rhizomes of *P. kurrooa*: (a) *P. kurrooa* rhizomes obtained from Dewal, Chamoli (reddish brown in colour), (b) rhizomes from Ukhimath, Rudraprayag (thicker and dark brown in colour), and (c) rhizomes from Darma valley, Pithoragarh (light brown in colour and strong aroma).

Figures 2 and 3 show the study area and images of *P. kurrooa* rhizomes samples obtained from different locations, respectively.

### 2.2. Preparation of the Plant Extract

Plant materials were cleaned off the rootlets and unwanted material. To remove any moisture that might have accumulated during transportation, the plant materials were kept at room temperature for air drying till the constant dry weight is attained [15]. The dried materials were powdered in an electronic grinder. 25 gm of powdered root was successively extracted with 150 ml of methanol, ethanol, and aqueous as solvents using the Soxhlet extraction method for 72 hours each [13]. After complete extraction, the extracts were filtered through Whatman filter paper no.1 and then transferred into sterile dry Petri plates and kept at room temperature until the solvent evaporated. The extracts were then scrapped out from Petri plates and preserved in airtight screw cap vials and kept in refrigerators at 4-5°C for further use [40].

### 2.3. Biochemical Profile of the Extracts

#### 2.3.1. Estimation of the Total Phenolic Content (TPC)

**Figure 4:**
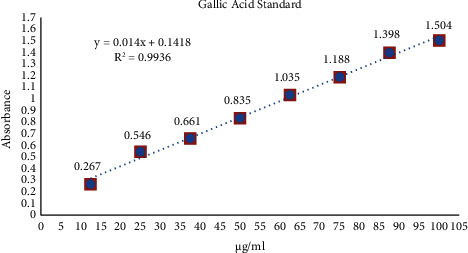
Gallic acid standard curve.

The Folin–Ciocalteau method was used to determine the TPC from the rhizomes (Singleton and Rossi) [41]. The stock solution was created by dissolving 10 mg of the extract in 10 ml of distilled water. This stock solution was centrifuged at 5000 rpm for 15 minutes. Out of this stock's supernatant, a set of 100, 200, and 300 *µ*g/ml solutions were prepared. 0.5 ml from each solution was mixed with 5 ml of the Folin–Ciocalteau reagent (diluted by adding 20 ml of FC reagent in 200 ml DW), and the reaction was then allowed to complete for 7 minutes at room temperature. To stop the reaction, 4 ml of 7.5% of sodium carbonate solution was added and blue colour was developed. After an hour of incubation, absorbance was measured in a UV-vis spectrophotometer at 765 nm against a blank (created with DW). Using a regression equation derived from the gallic acid standard curve (Figure 4), the TPC was calculated (as g/ml equivalent gallic acid).

#### 2.3.2. Total Tannin Content

The Folin–Ciocalteu method was used to determine the tannin content. The stock solution was created by dissolving 10 mg of the extract in 10 ml of distilled water. In test tubes with 7.5 ml of distilled water, a set of 20, 40, 60, 80, and 100 g/ml solutions were made from this stock (each with three technical replicates). A 0.5 ml dose of the Folin–Ciocalteu reagent was added to the solutions. After a few minutes, 1 ml of 35% sodium carbonate solution was added. The solutions were then diluted to 10 ml with distilled water. After thoroughly shaking, this mixture was left at room temperature for 30 minutes. UV-vis spectrophotometer at 700 nm was used to measure the absorbance of the test and standard solutions against a blank. The blank was prepared using distilled water instead of the rhizome extract.

**Figure 5:**
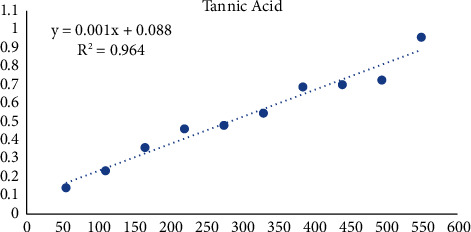
Tannic acid standard curve.

Equations derived from the tannic acid standard (Figure 5) were used to calculate the sample's total tannin content and express as mg of tannic acid equivalents (TAEs) per gram of dry weight of the extract.

#### 2.3.3. Total Flavonoid Content (TFC)

**Figure 6:**
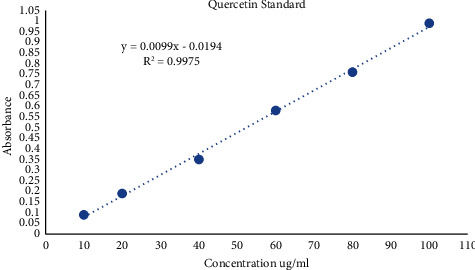
Quercetin standard curve.

The method given by Zhishen et al. [42] was used to determine the total flavonoid content. About 3 mg of the extract was dissolved in 10 ml of methanol to make a stock solution; out of this stock, 300 ml was taken in a test tube to which 3.4 ml of aqueous methanol (30%), 150 ml of sodium nitrite of 0.5 M (0.425 mg NaNO_2_ in 10 ml DW), and 150 ml of aluminium chloride solution of 0.3 M (400 mg AlCl_3_ in 10 ml DW) were added. After 5 minutes of incubation at room temperature, we added 1 ml of sodium hydroxide solution of 1 M (4 gm NaOH in 100 ml DW). The experiment was performed in technical triplicates. Absorbance was measured against a blank (made using DW) at 506 nm in a UV-vis spectrophotometer. The total flavonoid content was calculated as mg quercetin/ml using an equation derived from the quercetin standard curve (Figure 6).

#### 2.3.4. Total Alkaloid Content

The method given by Shamsa et al.[43] for the determination of alkaloids was used. About 50 mg of the methanolic extract of rhizomes was dissolved in 10 ml of 2 N HCl (made by dissolving 16.423 ml HCl in 25 ml DW and adjusting the final volume to 100 ml with distilled water) and was filtered using Whatman no.1 filter paper. To the separatory funnel, 1 ml of the filtrate was transferred and further washed three times with 10 ml of chloroform. The solution's pH was adjusted to 7.0 using 2 N NaOH (8 gm of NaOH dissolved in 100 ml DW).

**Figure 7:**
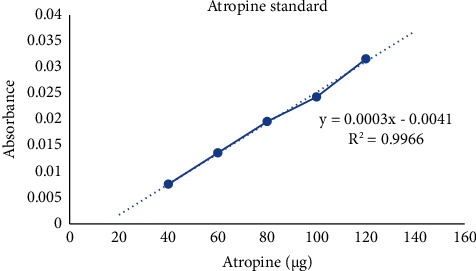
Atropine standard curve.

Adding 5 ml of bromocresol green (BCG) solution (by dissolving 69.8 mg of BCG in 3 ml of 2 N NaOH and 5 ml of distilled water and once entirely dissolved, the volume is made up to 1000 ml) and 5 ml phosphate buffer (made by dissolving 7.16 g of sodium phosphate monobasic monohydrate in 100 ml distilled water) to the solution, the mixture was briskly shaken with 1, 2, 3, and 4 ml of chloroform in a sequential process. The collected extract was diluted with chloroform to make up the volume up to 10 ml. The experiment was performed in technical triplicate. The absorbance of the extracts was measured using the UV-vis spectrophotometer at 470 nm. The amount of total alkaloid in the extracts was estimated using a standard curve created with various atropine concentrations (Figure 7).

### 2.4. Antioxidant Activity of the Extracts

#### 2.4.1. Determination of the Antioxidant Activity Using the 2,2-Dipehnyl-1-Picrylhydrazyl (DPPH) Radical Scavenging Method

The method proposed by Katalinic et al. [44] was used to evaluate the antioxidant activity of the plant extracts against DPPH, with slight modifications. A methanolic dilution DPPH of 1 × 10^−4^ M (4 mg DPPH in 100 ml methanol) was prepared. The stock solution was made with extracts in different concentrations, i.e., 0.1, 0.5, and 1 mg/ml mother solvent. 1 ml aliquots of each stock (two replicates per sample) were dissolved with 2 ml of methanolic dilution of DPPH. After 16 minutes of dark storage at room temperature (37°C), the mixture's absorbance was recorded using the UV-vis spectrophotometer at 517 nm. The blank was prepared with methanol. The following formula was used to determine the antioxidant activity and percentage:

(1) $$\begin{array}{c} \%\text{ Antioxidant activity}=\frac{\left(\text{AC}—\text{AS}\right)}{\text{AC}}\times 100, \end{array}$$

where AC = absorbance of the control and AS = absorbance of the sample.

#### 2.4.2. Estimation of Antioxidant by the Reducing Power Assay

The method outlined by Chu et al. and Quy et al. [45, 46] with slight adjustment was used to evaluate the reducing power of rhizome extracts. 10 mg of the extract was dissolved in 10 ml of the mother solvent to make the stock solution. In a test tube, 2.5 ml of the stock solution and 2.5 ml of sodium phosphate buffer of 0.2 M and pH 6.6 (made by dissolving 3.561 gm of Na_2_HPO_4_ in 100 ml DW and 2.76 mg of NaH_2_PO_4_·H_2_O in 100 ml DW and then mixing 77 ml of the dibasic solution and 23 ml of the monobasic solution) were added and, subsequently, added 2.5 ml of potassium ferricyanide (1% = 20 gm in 200 ml DW) and mixed well. The mixture was incubated in the water bath for 20 minutes at 50°C. After removing the tubes from the water bath, 2.5 ml of trichloroacetic acid (10% = 20 ml TCA in 180 ml DW) was added to the mixture and centrifuged for 10 minutes at 3000 rpm. 1.25 ml of distilled water and 25 *µ*l of ferric chloride (0.1% = 100 mg FeCl_3_ in 100 ml DW) were added to 125 *µ*l of the supernatant in a test tube. The content of the tube was well mixed by shaking with hands, and the absorbance was measured using a UV-vis spectrophotometer at 700 nm. The absorbance of the control was 0.58.

### 2.5. Statistical Analysis

Data were expressed as the mean ± standard deviation (SD). One-way ANOVA in Microsoft Excel was used to analyze the significant differences among the samples.

## 3. Results and Discussion

The present investigation entitled “Comparative study on biochemical profile and antioxidant activity of *Picrorhiza kurrooa* obtained from three different districts of Uttarakhand” was conducted at HAPPRC, HNBGU Srinagar Garhwal, Uttarakhand, India, during the year 2022. The findings of the present study are presented in this chapter under following headings.

### 3.1. Total Phenolic Content (TPC)

**Figure 8:**
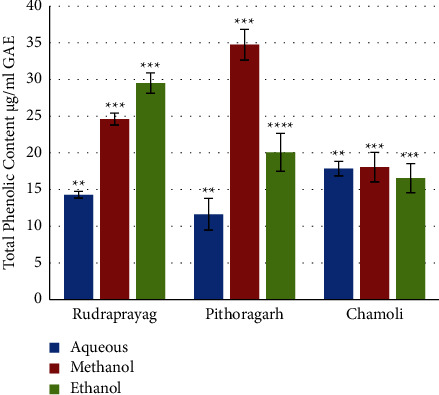
Variation in the total phenolic content in different extracts of *P. kurrooa*. Data are expressed as the mean values ± SD. *p* values ^*∗*^ <0.05, ^*∗∗*^ <0.01, ^*∗∗∗*^ <0.001, ^*∗∗∗∗*^ <0.0001, and *N* = nonsignificant when compared among the aqueous extracts, methanol extracts, and ethanol extracts of *P. kurrooa* collected from the three sites.

The results from one way ANOVA showed that the TPC was significantly affected by location factors. The total phenolic content was found to be at the maximum level in the methanol extract of *P. kurrooa* of Pithoragarh, i.e., 34.74 ± 2.1 *μ*g/ml GAE, followed by the ethanolic extract of Rudraprayag, i.e., 29.50 ± 1.30 *μ*g/ml GAE, while the minimum was observed in the aqueous extract of Pithoragarh, i.e., 11.62 ± 2.17 g/ml GAE. Results of spectrometric analysis are illustrated in Figure 8. One way ANOVA results revealed that the TPC in the aqueous extract of rhizome of Chamoli was significantly higher than Rudraprayag and Pithoragarh (*p* = 0.0044 and Fischer's LSD = 2.23), the TPC in methanolic extracts of rhizomes was significantly higher in Pithoragarh than Rudraprayag and Chamoli (Fischer's LSD = 1.17 and *p* = 0.00019), and the TPC in ethanolic extracts of rhizomes was significantly higher in Pithoragarh as compared to Chamoli and Rudraprayag (*p* = 0.00062 and Fischer's LSD = 4.99).

### 3.2. Total Tannin Content

**Figure 9:**
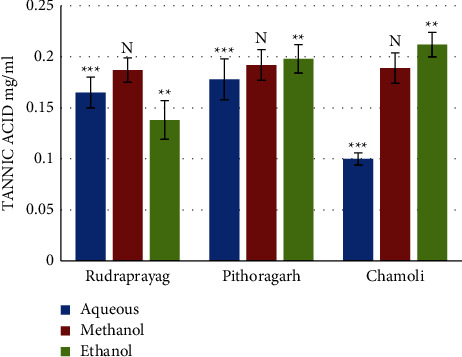
Total tannin content in different extracts of *P. kurrooa.* Data are expressed as the mean values ± SD. *p* values ^*∗*^ <0.05, ^*∗∗*^ <0.01, ^*∗∗∗*^ <0.001, ^*∗∗∗∗*^ <0.0001, and *N* = nonsignificant when compared among the aqueous extracts, methanol extracts, and ethanol extracts of *P. kurrooa* collected from the three sites.

The total tannin content was found to be highest in the ethanolic extract of *P. kurrooa* obtained from Chamoli (0.212 ± 0.012 mg/ml TAE), followed by the ethanolic extract of Pithoragarh (0.198 ± 0.015 mg/ml TAE). The minimum total tannin content was 0.10 ± 0.006 mg/ml TAE found in the aqueous extract of *P. kurrooa* of Chamoli district. Results of spectrometric analysis are shown in Figure 9.

One way ANOVA results indicate that the total tannin content (TTC) in the aqueous extract of rhizomes from Pithoragarh was significantly higher than Rudraprayag and Chamoli (*p* = 0.0010 and Fischer's LSD = 0.079); one way analysis results for the TTC in the methanolic extract were nonsignificant, and the TTC in the ethanolic extract of rhizomes from Chamoli was significantly higher than Pithoragarh and Rudraprayag (*p* = 0.0064 and Fischer's LSD = 0.068).

### 3.3. Total Flavonoid Content (TFC)

**Figure 10:**
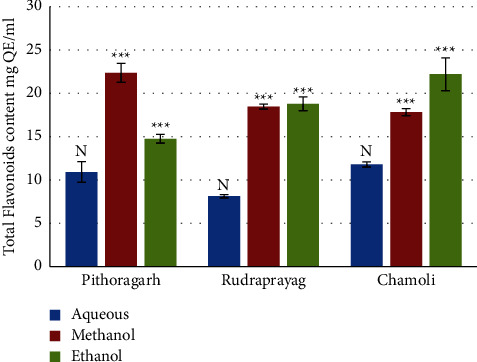
Total flavonoid content in extracts of *P. kurrooa.* Data are expressed as the mean values ± SD. *p* values ^*∗*^ <0.05, ^*∗∗*^ <0.01, ^*∗∗∗*^ <0.001, ^*∗∗∗∗*^ <0.0001, and *N* = nonsignificant when compared among the aqueous extracts, methanol extracts, and ethanol extracts of *P. kurrooa* collected from the three sites.

The highest flavonoid content was shown by the methanolic extract of *P. kurrooa* from Darma valley, Pithoragarh district, i.e., 22.96 ± 0.21 *μ*g QE/ml, followed by the ethanolic extract of Chamoli, i.e., 22.31 ± 1 *μ*g QE/ml. The least flavonoid content was found to be 8.12 ± 0.2 *μ*g QE/ml. Results are illustrated in Figure 10.

One way ANOVA results for the total flavonoid content revealed that results for aqueous extracts of rhizomes from 3 sites were nonsignificant; in the methanolic extract, the TFC was significantly higher in rhizomes from Pithoragarh as compared to that of Chamoli and Rudraprayag (*p* = 0.00034 and Fischer's LSD = 0.016), and the TFC in the ethanolic extract of rhizome from Chamoli was significantly higher than that of Rudraprayag and Pithoragarh (*p* = 0.00087 and Fischer's LSD = 0.029).

### 3.4. Total Alkaloid Content (TAC)

**Figure 11:**
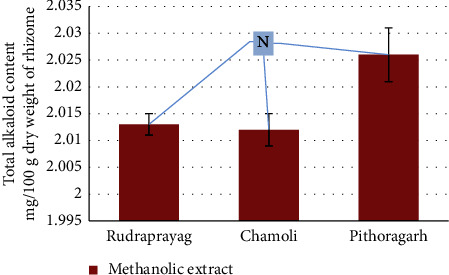
Variation in the total alkaloid content in methanolic *P. kurrooa*. Data are expressed as the mean values ± SD. *p* value *N* = nonsignificant when compared among the aqueous extracts, methanol extracts, and ethanol extracts of *P. kurrooa* collected from the three sites.

The methanolic extract of the *P. kurrooa* sample from Pithoragarh showed the highest amount of alkaloid content, i.e., 202.6 ± 2.2 mg/100 g dry weight of rhizome, and the methanolic extracts of the Chamoli and Rudraprayag samples showed almost the same alkaloid content (201.2 ± 3.5 mg/100 g dry weight of rhizome and 201.3 ± 8 mg/100 g dry weight of rhizome, respectively). Results are illustrated in Figure 11. One way ANOVA results for the total alkaloid content in the methanolic extract of rhizomes were nonsignificant.

### 3.5. Evaluation of the Antioxidant Activity Using the 2,2-Dipehnyl-1-Picrylhydrazyl (DPPH) Radical Scavenging Method

**Figure 12:**
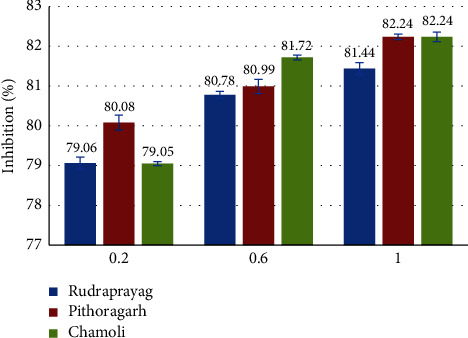
Inhibition% showed by the extracts of *P. kurrooa*.

The methanolic extract of the *P. kurrooa* sample from Pithoragarh showed the highest inhibition %, i.e., 82.24 ± 1.2%, followed by the methanolic extract of Chamoli, i.e., 82.14%. The least inhibition % was reported in the ethanolic extract of Chamoli, i.e., 47.24 ± 1.4%, at 1 mg/ml concentration of the extracts as illustrated in Figure 12 and Table 1.

### 3.6. Estimation of the Antioxidant Activity by Reducing Power Assay

**Figure 13:**
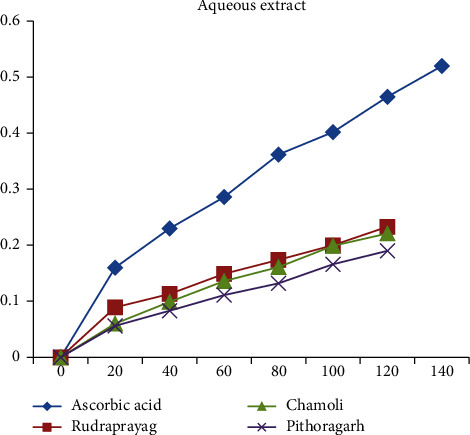
Reducing power showed by the aqueous extract of *P. kurrooa* against the ascorbic acid standard.

**Figure 14:**
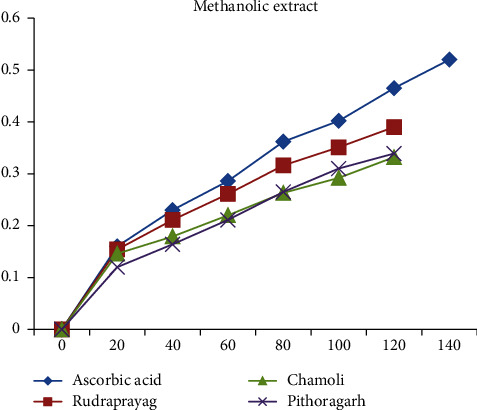
Reducing power showed by the methanolic extract of *P. kurrooa* against the ascorbic acid standard.

**Figure 15:**
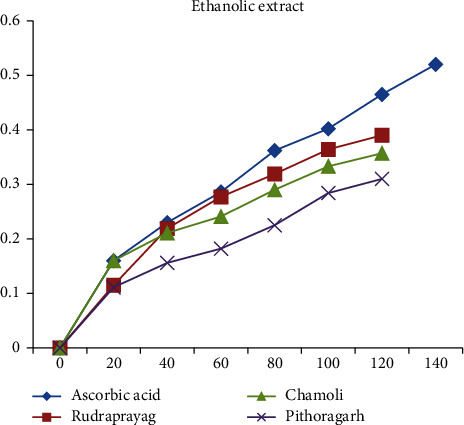
Reducing power showed by the ethanolic extract of *P. kurrooa* against the ascorbic acid standard.

When compared with the standard ascorbic acid, aqueous, methanolic, and ethanolic extracts of *P. kurrooa* obtained from Rudraprayag showed the highest reducing power, i.e., 0.233, 0.39, and 0.39, respectively, at the concentration 120 *μ*l, than the extracts of Pithoragarh and Chamoli, as shown in Figures 13–15.

## 4. Discussion

Plants are the source of numerous valuable drugs of natural origin. Analysis of elements and phytochemicals in edible plants plays a decisive role in assessing their nutritional significance. An herbal drug can be utilized as a medicinal agent only if it is authentic and bears acceptable standards and quality. Any illness cannot be managed effectively until the supply of a genuine drug sample is ensured. Therefore, before using a crude drug, a proper authentication method should be carried out for each crude drug, such as chemical study and anatomical, morphological, and organoleptic study [47].

The abovementioned parameters—total phenols, alkaloids, flavonoids, tannin content, and antioxidant potential—were assessed in the current study using spectrophotometric methods, and it was discovered that *Picrorhiza kurrooa* rhizomes from the Darma valley in the Pithoragarh district of the Kumaun region had the highest concentration for all the parameters.

The results obtained showed that all three *Picrorhiza kurrooa* samples possess high variation in all these metabolites. Based on the analysis, it was found that the total phenolic content, flavonoid content, total alkaloids, and DPPH radical scavenging activity of *Picrorhiza kurrooa* obtained from Darma valley were found to be the highest followed by Chamoli and least in Rudraprayag, while the highest tannin content was found in Chamoli and total reducing power was the highest in *Picrorhiza kurrooa* obtained from Rudraprayag.

Gunji village lies 3400 masl; in winter, temperature dips to −4°C, and in summer, the temperature in Gunji hovers around 10°C. Ghes village of Chamoli (2566 masl) is a moist temperate area. Average day time temperature in winters for Ghes is −3°C, and for summers, it is 18°C. In Pothiwasa, average day temperature during winters is −3°C and average temperature in summers is around 24°C [48]. Since the altitude of the three locations from where *Picrorhiza kurrooa* were obtained by the traders are different, i.e., Gunji (3400 masl), Ghes (2566 masl), and Pothiwasa (2200 masl), and with an altitude change, changes in various other factors such as ecological niche, temperature, UV radiation, and biotic and abiotic factors have also been reported [49]. Therefore, variation in the phytochemical content in all the samples may be due environmental factors. Prior research has also revealed that a variety of environmental parameters, including mean temperature, rainfall, harvest maturity, soil type, UV radiation, sunlight, and habitat conditions, may also have an impact on the formation and accumulation of secondary metabolites in plants [50, 51]. The findings of our study indicate that the *Picrorhiza* rhizomes from Gunji, located at an altitude of 3400 m above the sea level, possess a greater amount of biochemical compounds. These results are consistent with the research conducted by Pandit et al. in 2018, where they discovered that the concentration of picrosides in *Picrorhiza kurroa* Royle ex Benth. was significantly higher in specimens collected at higher altitudes compared to those collected at lower altitudes [52]. Earlier many reviewers have reported that the synthesis of the phenol and flavonoid contents in the plant increases with the increase in UV radiation at higher altitude [53, 54]. A previous study done by Katoch et al. suggests that these differences can also be due to the occurrence of genetically different strains collected from geographically different locations [55]. In addition, the age of the plant at the time of harvesting plays a significant role in determining SM accumulation. To obtain the highest levels of active constituents, *Picrorhiza kurrooa* is typically harvested after completing its reproductive and active growth phase, which generally occurs in September–October. Notably, a study conducted by Pandit et al. in 2013 observed that the rhizomes from three-year-old plants exhibited the highest concentration of active compounds [52].

Moreover, the duration and conditions of storage can significantly impact the secondary metabolite content in various parts of the plant. Therefore, it is imperative to follow specific guidelines for the preservation of *P. kurrooa*. This involves drying the plant material in the shade at a temperature of 15–20°C until it reaches a moisture content of approximately 8%. Subsequently, it should be stored in a dry environment at room temperature to preserve its distinctive aroma and phytochemical content [56].

There was a negative correlation of TRP and tannins with the phenolic content as it was observed maximum in rhizomes from Rudraprayag, western Himalayas, although many reports have revealed that there is a direct correlation between antioxidant activities and reducing power as well as tannins of certain plant extracts [57, 58]. The results of this study are supported by previous studies. However, studies conducted by Kähkönen et al. [59] on some plant extracts containing phenolic compounds found that there is no correlation between the phenolic content and tannins as well as TRA. The previous studies suggest that there is no correlation associated among phenolics, tannins, and TRP [60].

## 5. Conclusion

The results of this study indicated that *P. kurrooa* rhizomes obtained from Darma valley, Pithoragarh district (Kumaun region), are the richest sources of phenolic, flavonoids, alkaloids, and radical scavenging activity in comparison to the rhizomes obtained from the regions of Chamoli district and Rudraprayag district and therefore could serve as a basis for future drugs and food materials.

The phytochemical contents in *P. kurrooa* samples may have been influenced by various environmental factors such as climatic conditions, growing region, cultivation methods, harvesting time, and storage condition.

This study thus recommends that organizations involved in the manufacturing of pharmaceutical products should purchase *P. kurrooa* rhizomes (kutki) of high quality from the market while taking into account the concentration of phytochemical components for the development of pharmaceutical medications with desired efficacy. Since the *P. kurrooa* rhizomes of Darma valley, Pithoragarh, had a high quantity of most phytochemicals, hence this study also suggests that farmers/growers obtain/buy *Picrorhiza kurrooa* seeds and rhizomes from this region for propagation and multiplication to get better returns in the market and for reducing the need for unplanned collection. Based on our results, the author also proposes further exploration of medicinal plants in the Gunji, Darma valley, and other neighboring regions with the aim to identify and study similar high-value medicinal plants that hold the potential to provide significant benefits to the society. Advanced molecular tools and comparative phytochemical anatomical screening practices against adulteration-related issues can play a vital role in bioprospecting studies of this plant. *P. kurrooa* from Gunji, Pithoragarh, can be clinically tested for control and treatment of various diseases as well as for its toxicity. The potential pharmacological properties of *P. kurrooa*, such as its antimicrobial and antiviral activities, could be further investigated through the application of nanotechnology.

## Figures and Tables

**Table 1:** Inhibition % showed by different extracts of *P. kurrooa* at different concentrations.

Table	Rudraprayag			Pithoragarh			Chamoli		
Conc.	Aqueous	Methanol	Ethanol	Aqueous	Methanol	Ethanol	Aqueous	Methanol	Ethanol
0.2 mg/ml	14.8 ± 1.4	79.06 ± 1.5	10.17 ± 5.2	15.51 ± 2.4	80.89 ± 1.9	12.93 ± 1.2	18.95 ± 2.4	79.05 ± 0.1	12.24 ± 2.1
0.6 mg/ml	51.72 ± 3.05	80.78 ± 0.5	32.02 ± 1.8	64.40 ± 0.8	80.99 ± 1.8	35.6 ± 3	75.17 ± 0.23	81.72 ± 0.58	41.9 ± 0.9
1 mg/ml	62.91 ± 3.6	81.06 ± 1.9	52.93 ± 1.7	70.17 ± 3.2	**82.24** **±** **0.7**	58.72 ± 1.7	74.22 ± 1.3	82.14 ± 1.2	**47.24** **±** **1.4**

Bold values represent highest and the lowest values under 1 mg/ml concentration. Methanol extract of rhizomes from Pithoragarh showing highest and ethanol extract of rhizomes from Chamoli showing lowest.

## Data Availability

The data used to support the findings of the study are included within the article.
